# Death of the Late Dr. H. Neill

**Published:** 1846-01

**Authors:** 


*Death of the late Dr. H. Neill.*

In our own immediate circle of professional worthies, a painful void has recently been caused by the death of Dr. Henry Neill, who, if less known to literary and scientific fame than some of his brethren, has left behind him a well merited reputation for a life spent in the unostentatious exercise of the Christian virtues, and the patient, conscientious, and successful discharge of his duties as a physician, in full practice.

We forbear at this time from a detailed exhibition of the distinctive merits of the deceased,—as occasion will be offered, hereafter, for our doing so in a suitable manner, when availing ourselves of the information collected by the venerable President of the College of Physicians, who has been nominated to this trust by that body of which Dr. Neill had been for a long time Vice President.—*Bull. of Med. Sci.*

